# Commentary: Acceptance and commitment therapy combined with usual care improves psychosocial outcomes and reduces complications in patients with permanent colostomies after colorectal cancer surgery: a retrospective cohort study

**DOI:** 10.3389/fsurg.2026.1762530

**Published:** 2026-03-17

**Authors:** Yuan Luo, Xingxing Tao, Shuoyang Xu, Wenjiang Wu

**Affiliations:** Shenzhen Hospital (Futian) of Guangzhou University of Chinese Medicine, Shenzhen, Guangdong, China

**Keywords:** acceptance and commitment therapy (act), permanent colostomies, quality of life, retrospective cohort study, stoma-related complications

## Introduction

Shi et al. (1) retrospectively investigated the effects of integrating Acceptance and Commitment Therapy (ACT) with usual care for patients with permanent colostomies following colorectal cancer surgery, reporting improvements in psychosocial outcomes and a reduction in complications. Research on structured psychosocial interventions like ACT for this specific population remains limited. This commentary examines the methodological strengths and limitations of this retrospective study, which provides preliminary evidence for the potential benefits of ACT. It further discusses the clinical implications of the findings and identifies key directions for future research needed to substantiate them.

## Methodological considerations and strengths

The authors adeptly employ propensity score matching (PSM) to mitigate selection bias and achieve balanced groups—a notable strength. The use of linear mixed models appropriately captures temporal trends, suggesting ACT‘s progressive benefits. However, several limitations inherent to the retrospective design warrant caution. First, the study cannot establish causality, as unmeasured variables (e.g., baseline psychological flexibility or social support) may confound the results. Furthermore, potential recruitment bias cannot be ruled out; patients who elected to participate in ACT may have differed in unmeasured ways (e.g., higher baseline motivation) from those receiving usual care alone. Additionally, key aspects of the intervention and control conditions lack detailed specification: variations in the content and quality of ‘usual care' likely acted as an unmeasured confounder, and the study provides no details on how fidelity to the ACT protocol was ensured or measured, which could significantly affect the observed effect sizes and complicate replication. While PSM reduces overt bias, it cannot address such latent factors. Moreover, the single-center setting and small sample size (*n* = 120 after matching) limit the generalizability of the findings. Finally, although the handling of missing data via multiple imputation assumes data are missing at random, sensitivity analyses exploring alternative mechanisms would have strengthened the validity of the results. For future clinical translation, pragmatic trials comparing ACT delivered by nurses vs. specialists could better assess scalability while monitoring fidelity.

### Clinical implications and mechanistic insights

The large effect sizes (Cohen‘s d≈0.9) observed for improvements in self-efficacy, resilience, and quality of life suggest a substantial association worthy of further investigation, suggesting that ACT's focus on acceptance and values-based actions resonates deeply with stoma-related distress. This underscores the potential value of formalized psychological support beyond standard education or informal peer interaction, though the relative efficacy and optimal delivery mode (e.g., therapist-led ACT vs. facilitated peer support) warrant further comparison. The use of validated stoma-specific instruments, such as the Ostomy Adjustment Scale (2), could further enhance the precision of outcome measurement in future studies. The correlation analyses revealing strong links between these outcomes (e.g., r = 0.72 for self-efficacy and QoL) provide a plausible mechanism: enhanced self-efficacy may drive better stoma care adherence, indirectly reducing complications like dermatitis. This aligns with ACT's theoretical framework, where psychological flexibility fosters adaptive behaviors. However, the study's inability to delineate temporal precedence among variables—does self-efficacy boost resilience or vice versa?—leaves mechanistic pathways incompletely mapped. To address this, future studies could employ longitudinal designs with time-lagged analyses to establish temporal precedence, or use dynamic assessment methods like Ecological Momentary Assessment to capture real-time interactions and test mediation effects. On a clinical level, integrating ACT into stoma care protocols via brief group sessions could maximize reach, though its comparative effectiveness against other support modes requires further evaluation. The reduction in complications, though statistically significant, should be interpreted cautiously due to the observational design; for instance, dermatitis rates may reflect unmeasured nursing care quality rather than ACT alone. Future research could explore mind-body interactions in this population by, for example, integrating patient-reported outcomes with objective biomarkers such as cortisol levels.

### Future directions and concluding discussion

In summary, Shi et al. (1) make a compelling case for ACT's role in stoma care, but their findings should catalyze more rigorous investigations. Beyond RCTs, hybrid effectiveness-implementation designs are needed. These studies should evaluate ACT‘s real-world uptake while measuring contextual factors like organizational readiness. Mechanistically, linking psychosocial gains to biomarkers (e.g., cortisol levels) could unveil preliminary mind-body pathways, informing personalized interventions. A pivotal next step involves RCTs comparing ACT with other therapies (e.g., cognitive-behavioral therapy) to establish comparative effectiveness, while economic evaluations could assess cost-effectiveness in real-world settings. The progressive improvement pattern over time hints at ACT's long-term benefits, warranting extended follow-ups beyond 6 months to evaluate sustainability. Importantly, subgroup analyses based on baseline distress levels or coping styles could help personalize interventions. This approach would maximize impact for the most vulnerable individuals. While this study excels in highlighting psychosocial gains, it also reminds us that retrospective designs are stepping stones rather than endpoints; embracing prospective, methodologically diverse approaches will be key to advancing evidence-based care. Ultimately, the integration of psychological support into routine stoma management, as advocated here, holds immense potential, but it must be grounded in robust science to ensure equitable and lasting benefits for patients navigating the complexities of life with a permanent stoma.
